# Supplementary material for: Investigating the influence of physiologically relevant hydrostatic pressure on CHO cell batch culture
Source: Sci Rep. 2021 Jan 8;11:162. doi: 10.1038/s41598-020-80576-8 (PMC7794228; doi:10.1038/s41598-020-80576-8)
Supplement: Supplementary file 1 — Supplementary Information 1. [file 41598_2020_80576_MOESM1_ESM.zip › S1/IgG/Feb17 60 90 standard/old/Feb13 60mmHg 3.pdf]

===

Acq. Operator : SYSTEM   Seq. Line : 26
Sample Operator : SYSTEM
Acq. Instrument : HPLC-0039   Location : 2
Injection Date : 2/17/2019 3:52:12 PM   Inj : 1
Inj Volume : 100.000 µl
Method : C:\Users\Public\Documents\ChemStation\1\Data\TK_CHO_IgG 2019-02-17 14-04-01\CHO_IgG_LC.M (Sequence Method)
Last changed : 2/8/2019 3:17:23 PM by SYSTEM

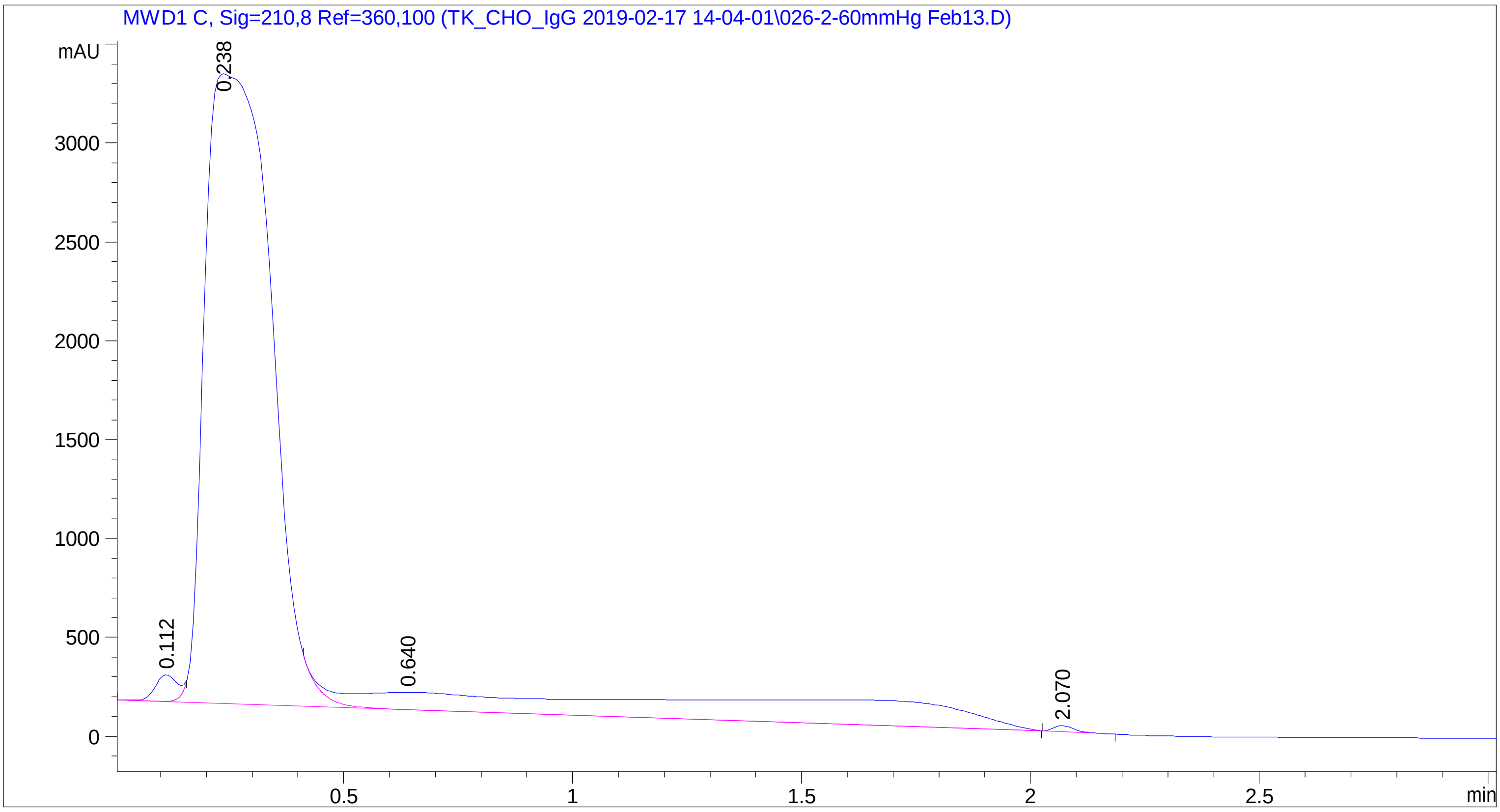

===

Area Percent Report

===

Sorted By : Signal
Multiplier : 1.0000
Dilution : 1.0000
Use Multiplier & Dilution Factor with ISTDs

Signal 1: MWD1 C, Sig=210,8 Ref=360,100

Peak #	RetTime [min]	Type	Width [min]	Area [mAU*s]	Height [mAU]	Area %
1	0.112	BV E	0.0497	437.82767	135.61301	1.0705
2	0.238	VV R	0.1332	3.20167e4	3185.85059	78.2811
3	0.640	VB E	1.1557	8352.67969	88.13573	20.4223
4	2.070	BBA	0.0481	92.47322	29.90834	0.2261

Totals : 4.08997e4 3439.50766

===

*** End of Report ***
